# Silver nanowires as plasmonic compensators of luminescence quenching in single up-converting nanocrystals deposited on graphene

**DOI:** 10.1038/s41598-021-82699-y

**Published:** 2021-02-11

**Authors:** A. Prymaczek, M. Cwierzona, M. A. Antoniak, M. Nyk, S. Mackowski, D. Piatkowski

**Affiliations:** 1 Institute of Physics, Faculty of Physics, Astronomy and Informatics, Nicolaus Copernicus University, Grudziądzka 5, 87-100 Toruń, Poland; 2 Advanced Materials Engineering and Modelling Group, Faculty of Chemistry, Wroclaw University of Science and Technology, Wybrzeże Wyspiańskiego 27, 50-370 Wroclaw, Poland

**Keywords:** Nanoscience and technology, Optics and photonics

## Abstract

Single nanocrystal spectroscopy is employed to demonstrate metal-enhanced optical response of Er^3+^/Yb^3+^ doped up-conversion nanocrystals deposited on graphene upon coupling with silver nanowires. Direct interaction between nanocrystals and graphene results in quenching of up-conversion emission and shortening of luminescence decay times, due to the energy transfer to graphene. The amount of the energy absorbed by graphene can be enhanced by coupling Er^3+^/Yb^3+^ doped up-conversion nanocrystals with silver nanowires. Microscopy studies with high spatial resolution together with time-resolved analysis of nanocrystal luminescence show increase of the emission rates with fourfold enhancement of the intensity for nanocrystals placed in the vicinity of silver nanowires. This strong enhancement emerges despite simultaneous interaction with graphene. The hybrid nanostructure provides thus a way to combine optical activity of up-conversion nanocrystals and enhancement provided by metallic nanowires with excellent electrical and mechanical properties of graphene.

## Introduction

Graphene, a monolayer of carbon atoms arranged into a two-dimensional hexagonal lattice, which exhibits high crystal quality, is well known for its remarkable thermal, mechanical, and electrical properties^1^. From the point of view of optical activity, a single layer of graphene absorbs 2.3% of incident light, independently of the wavelength, in the spectral range from 400 to 750 nm^2^. Although the zero-energy gap of graphene inhibits in principle any photoemission associated with radiative recombination of charged carriers, under specific circumstances, visible emission upon excitation with ultrashort laser pulses has been demonstrated^3^. Recently, an important body of new effects and applications is focused on hybrid nanostructures, combining unique properties of graphene and various nanoemitters.

Indeed, combination of functional molecules with excellent electrical properties of graphene opens possibility for new applications in sensorics and optoelectronics^4–7^. However, weak optical activity of graphene, including absorption and absence of band-to-band emission, makes it not very useful for strictly optical applications. To overcome this problem the concept of optical antennas (sensitizer) coupled with graphene has been postulated^8^. For instance, in the graphene-quantum dots coupled system, amount of energy absorbed by the antenna and efficiently transferred to graphene exceeds absorption possibilities of graphene itself^9^. It was shown that efficiency of the energy transfer depends also on the emission wavelength and on the number of graphene layers^10^. In these experiments graphene was recognized to be a perfect acceptor. For this reason, emission of the sensitizer within such a hybrid system is very strongly disrupted or can even be completely quenched^11^.

In this paper, we construct an advanced nanostructured material, which combines physico-chemical functionalities with optical and plasmonic properties, and experimentally study its capability for optimized optical response. The main components for engineering the novel range of functionalities of luminescent up-conversion nanoparticles prepared by solution wet chemistry methods concern combination of energy transfer and plasmon enhancement. In particular, we present a new type of three-component hybrid nanostructure consisting of graphene layer combined with rare-earth co-doped nanocrystals (NCs) and silver nanowires (NWs). Rare-earth doped materials are known for their outstanding optical properties, utilizing Stokes and anti-Stokes (up-conversion) luminescence occurring in the visible and infrared spectral ranges^12–14^. Due to unique optical properties they have found number of applications in lasers^15^, displays^16,17^, bio-medical contrasts/markers^18,19^ and sensors^20^. Recently, their application palette has been significantly extended due to incorporating interactions with graphene^21–25^. Herein, we use up-conversion NCs to sensitize sequential 2-photon absorption and introduce emissivity to the graphene-based hybrid system.

On the other hand, silver nanowires are known to enhance both Stokes and anti-Stokes luminescence of various emitters due to antenna and Purcell effects^26–28^. Due to their elongated shapes and lengths in the range of tens of micrometers, they facilitate transport of the energy through surface plasmon polaritons (SPPs), which has been considered in the context of applications in sensorics and integrated optoelectronic circuits^29–32^. Herein, we use silver nanowires to enhance efficiency of the NCs absorption/emission, and to compensate luminescence quenching that is usually observed in the presence of graphene^11^. Consequently, the three-component hybrid nanostructure features bright luminescence despite the interaction with graphene. We show that under certain conditions it is possible to observe simultaneous interactions between all three ingredients (graphene-nanocrystals-nanowires) within the nanostructure. The optical properties of such a system, which bridges all characteristic features of its components, i.e. spectrally broad absorption, up-conversion luminescence, sensitivity to light polarization, can be accompanied by excellent electrical conductivity and mechanical strength of graphene. Appropriate tailoring of the nanohybrid structure not only minimizes any interference between functionalities, but can also either bring synergy between them or impart a new property to the advance nanomaterials. Due to this combination, and the additional functionalities resulting from interactions between its components, the described hybrid nanostructure may find applications in sensorics and optoelectronics devices.

## Materials and methods

The hybrid nanostructure studied in this work is assembled of three components: a layer of graphene, rare-earth doped nanocrystals, and silver nanowires. High quality monolayer graphene was prepared by chemical vapor deposition, as described elsewhere^33^. In this process, graphene is grown on a copper substrate and then it is covered by a polymer layer. In the next step, the metallic substrate is etched chemically, and graphene is transferred on a glass coverslip. In the final step, the stabilizing polymer is dissolved in a suitable solvent. High quality graphene substrates used in this work were purchased in ACS materials.

**Figure 1:**
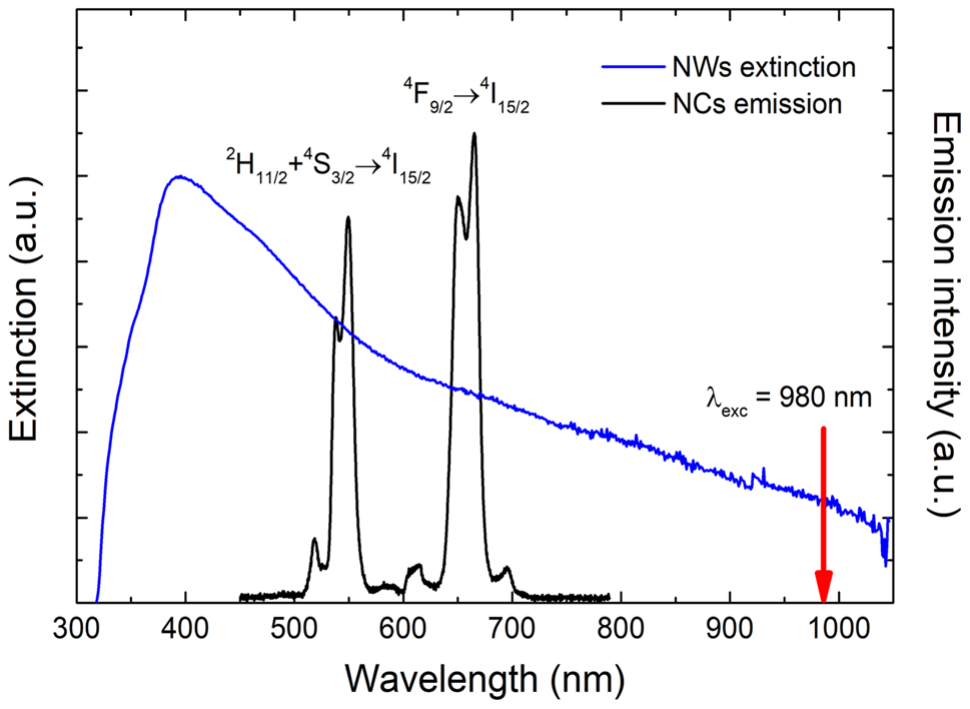
Extinction spectrum of a colloid of silver nanowires (blue line) together with the emission spectrum of a single nanocrystal (black line). The excitation of 980 nm laser diode was used, as indicated by the red line.

Silver nanowires were synthesized chemically using polyol process^34^. Their lengths vary from 10 to 15 µm, while their diameters are of the order of 100 nm. Extinction spectrum of Ag nanowires (Fig. 1) spans from about 350–1000 nm, and is mainly attributed to activation of surface plasmon oscillations^35^. Colloidal solution of silver nanowires was spin-coated on the graphene layer with the concentration optimized in such a way that on average 2–3 nanowires are deposited per 100 µm^2^ on the surface (Figure S1a, supplementary).

**Figure 2:**
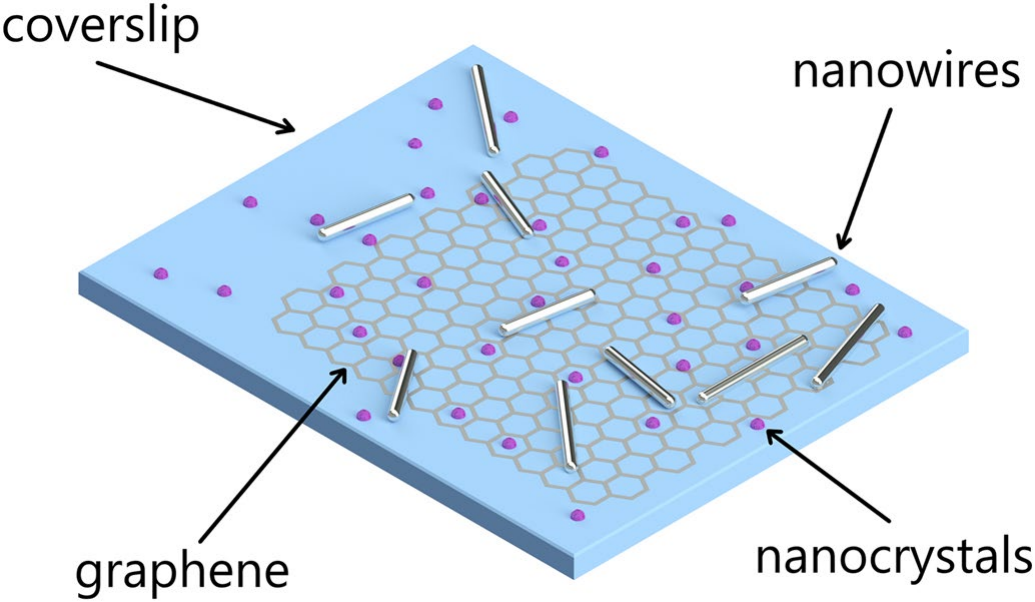
Illustratation of the examined sample (not to scale). Nanocrystals and silver nanowires deposited on graphene or glass are accesable.

For optical probing the interactions present in the hybrid nanostructure, we used up-converting NaYF_4_ NCs doped with Er^3+^ (2%) and Yb^3+^ (20%) ions, synthesized as described previously^36^. Transmission electron microscopy images indicate that the NCs are of quasi-spherical shape, with typical diameters of about 30 nm (Figure S1b, c)^37^. Upon excitation at 980 nm, the nanocrystals exhibit two-photon up-conversion luminescence at wavelengths of 540 nm and 660 nm (as shown in Fig. 1) assigned to ^2^H_11/2_,^4^S_3/2_ → ^4^I_15/2_ and ^4^F_9/2_ → ^4^I_15/2_ f-f electric-dipole transitions in Er^3+^, respectively^13^. Importantly, both absorption and emission of NCs overlap with the extinction spectrum of the NWs, therefore they can be potentially enhanced due to plasmonic effects^26^. The NCs concentration used in the experiments was of about 0.5 mg/ml. 10 μl of chloroform solution of NCs was spin-coated on the substrate (3000 rpm for 30 s) containing silver nanowires previously deposited on the graphene layer. Because graphene covers only part of the substrate, both isolated NCs, NWs, and coupled NCs-NW nanostructures deposited on graphene or glass can be accessible within one sample (Fig. 2).

Exemplary AFM images illustrating spatial organization of the nanoparticles are presented in Fig. S2. Obtained surface density of the NCs was about 4 per μm^2^ and the average distance between NCs exceeds resolution of the microscope. Therefore optical properties of individual NCs can be probed using typical confocal configuration.

The optical properties of the hybrid nanostructure were examined using confocal luminescence microscopy and spectroscopy. The experimental setup was based on a Ti-S microscope body (Nikon). The sample was mounted on a piezoelectric stage (PI-517, Physik Instrumente), allowing for raster-scanning along both *x* and *y* directions, and illuminated through an oil-immersion, high numerical aperture objective (Apo TIRF 60 × NA = 1.49, Nikon). For excitation we used continuous-wave/pulsed fiber coupled 10 mW single mode laser diode, operating at 980 nm. The laser beam is formed into a Gaussian beam, which allows to achieve excitation spot of about 450 nm in a diameter. Photoluminescence (PL) of NCs was detected using single photon counting module (SPCM-16, PerkinElmer) coupled with multifunctional counter card (PCI-6289, National Instruments). The images were acquired using band pass optical filters: 550/40 nm and 650/40 nm, dedicated for green and red emission of NaYF_4_:Er^3+^/Yb^3+^ nanocrystals, respectively. Time-resolved measurements (luminescence transients) were collected by a multiscaler card (MSA-300, Becker&Hickl) with resolution about 1 µs, while the laser was triggered by a programmable functional generator (Keithley 3390). The emission spectra were recorded with Czerny-Turner monochromator (Shamrock 500, Andor), coupled with CCD camera (Newton, Andor), and operating with the spectral resolution better than 1 nm.

## Results and discussion

The functionality of the hybrid nanostructure composed of three components: up-converting nanocrystals, silver nanowires and graphene, is determined by interactions that occur between the components in close proximity. As graphene is an efficient acceptor of energy, it is expected that upon excitation of the up-converting nanocrystals, the energy will be dissipated as heat in the graphene layer via non-radiative energy transfer. The absence of any luminescence from graphene implies that the energy transfer can be quantified exclusively by measuring the emission intensities and lifetimes of the NCs emission. On the other hand, placing the NCs in the vicinity of silver nanowires, which exhibit strong plasmon resonance, leads to enhancement of up-converted emission. As shown previously, the enhancement measured in such a structure originates from the combination of increase of the absorption and excitation rates (the Purcell effect) associated with the shortening of luminescence lifetime^26^.

**Figure 3:**
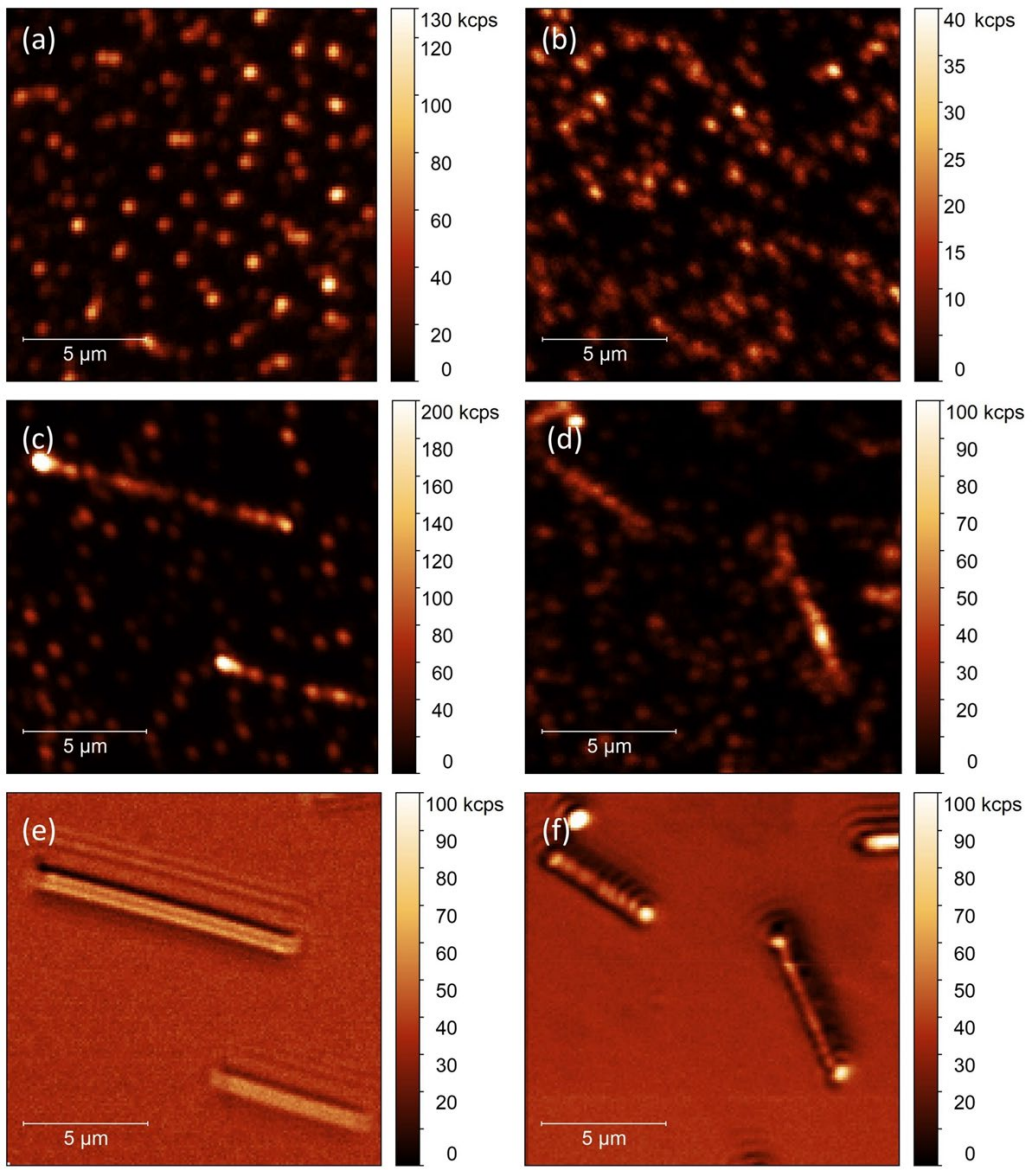
Photoluminescence intensity maps of NaYF_4_:Er^3+^/Yb^3+^ nanocrystals deposited on (**a**) glass and (**b**) graphene layer. Maps acquired for NCs coupled with silver nanowires placed on (**c**) glass and (**d**) graphene. Positions of nanowires on (**e**) glass and (**f**) graphene observed in a back scattering mode. Photoluminescence maps were collected at 550 nm under 980 nm laser excitation.

In Fig. 3 we depict typical luminescence intensity maps obtained for four samples: (a) up-converting nanocrystals deposited on a glass substrate, (b) up-converting nanocrystals deposited on graphene, (c) up-converting nanocrystals and silver nanowires on a glass substrate, and (d) up-converting nanocrystals and silver nanowires deposited on a graphene substrate. The excitation wavelength of 980 nm was used, and the emission was detected at 550 nm. In all images one can see well-defined, diffraction-limited spots, attributed to the luminescence of individual (isolated) NCs or small agglomerates thereof, placed randomly on the surface^38^. Rough comparison of the images obtained for graphene and glass substrates indicates that the intensities measured for the up-converting NCs deposited on graphene are considerably lower, presumably due to the non-radiative energy transfer from NCs to graphene. In addition, we notice twice as large surface density of nanocrystals on graphene with respect to the glass substrate, which might be due to better surface adhesion of the colloid to the graphene.

**Figure 4:**
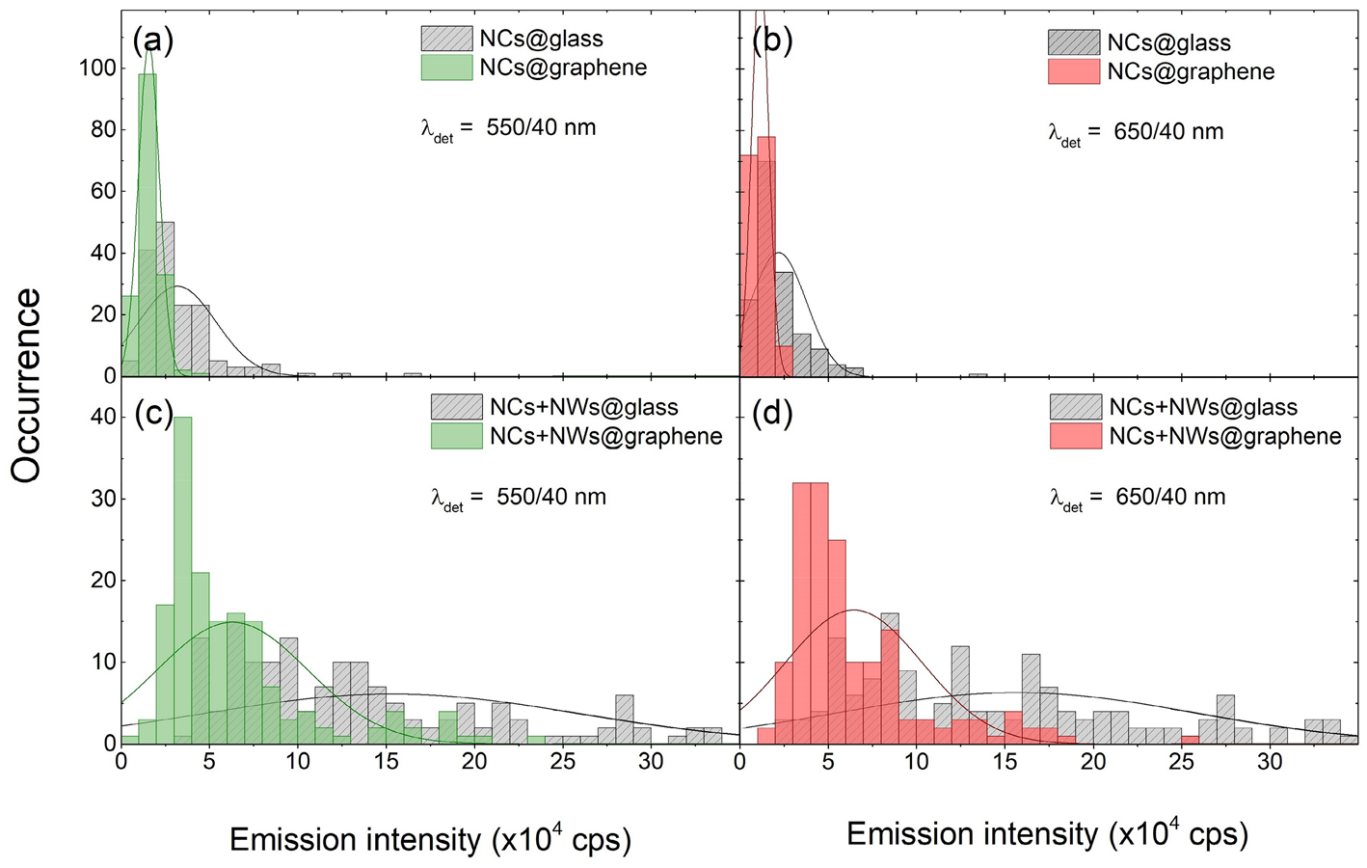
Histograms of photoluminescence intensity of (**a**,**b**) nanocrystals and (**c**,**d**) nanocrystals combined with silver nanowires, deposited on glass and graphene. Green (**a**,**c**) and red (**b**,**d**) emission of Er^3+^ is shown. Each histogram was obtained for approximately 150 individual NCs.

Statistical distributions of the emission intensity obtained for single nanocrystals deposited on glass and graphene are presented in Fig. 4 for both emission bands: (a) 540 nm and (b) 660 nm. The analysis was carried out for hundreds of single emission spots (for each type of the substrate), but these spots were preselected. The criteria concerned regular circular shape of the spot and diffraction limited size thereof in both directions. Such a procedure virtually guarantees that mostly single nanocrystals were taken into account. As compared with the reference (grey bars), upon depositing the nanocrystals on the graphene substrate, a clear delectable reduction of emission intensity is observed. On glass, for both emission lines intensities vary from 10 to 70 kcps, reflecting mainly the size dispersion of the nanocrystals^37^. Indeed, assuming homogeneous distribution of dopant ions within NCs and spherical shape thereof, seven-fold increase of the emission intensity translates into almost two-fold increase of the diameter. This simple calculations are consistent with TEM data, where observed diameters of the NCs vary from about 20–40 nm (Fig. S1b, c). Therefore, it should be noted that on a single nanocrystal level, the luminescence intensity depends on the number of Er^3+^ ions in the nanocrystal, which for this case vary from about 600–4800 ions^39^. Average intensity is about 30 kcps with the widths of the distributions (full width at half maximum) of about 40 kcps for both emission bands.

In the case of NCs deposited on graphene, the intensities range from 5 to 25 kcps for both emission bands, and are roughly 2 times lower than for NCs on glass, as can be seen in Fig. 4a,b (green and red bars for 540 nm and 660 nm emission, respectively). Indeed, the average intensity values are about 15 kcps for both emission bands, but the distributions are considerably narrower (approximately twofold) on graphene than on glass, amounting to 15 kcps. This observation suggests that NCs interact with graphene rather efficiently. Indeed, since the interaction between a single emitter and graphene is relatively long-ranged (∝ d^−4^)^40^, we can expect that the presence of graphene substrate affects most of the ions even within the largest (thus brightest) NCs, modifying their emission intensity^41,42^. On the other hand, the nonradiative energy transfer from NCs to graphene improves the amount of energy absorbed by the graphene itself. In other words, NCs sensitize graphene for IR sequential absorption of two photons.

While interactions between nanocrystals and graphene lead to significant reduction of the emission intensity, as reported also by others^11^, introducing plasmonically active metallic nanostructures may circumvent this effect, leading to a structure which remains optically active. The maps displayed in Fig. 3 obtained for hybrid nanostructures involving silver nanowires (Fig. 3c,d) feature emitters localized in the vicinity of silver nanowires (NWs) that exhibit strongly enhanced emission intensity. This increase is due to combination of enhanced absorption and emission rates, leading to the increase of the overall emission efficiency^27^. Importantly, the positions of silver nanowires can be precisely determined using a backscattering intensity maps shown in Fig. 3e,f for glass and graphene substrate, respectively.

Interaction between up-converting nanocrystals and silver nanowires strongly influences the distribution of emission intensity of the former. The results of statistical analysis presented in Fig. 4c,d show that average luminescence intensity of nanocrystals coupled to silver nanowires deposited on a glass substrate is about 160 kcps for both up-conversion emission lines. This implies over fivefold enhancement of luminescence intensity, which is comparable to previous results^26^. At the same time, the distribution of intensities is substantially broader compared to the reference. As metal enhanced luminescence is strongly dependent on the distance between an emitter and a metallic nanoparticle, variation of the distance directly translates into the variation of the emission intensity^38^.

Depositing both up-converting nanocrystals and silver nanowires on a graphene substrate turns on another interaction, namely, the energy transfer from the nanocrystals to graphene. It occurs for nanocrystals deposited and localized both in close to silver nanowires and in some distance from them. Exemplary luminescence intensity map is presented in Fig. 3d, and accompanied by the scattering image (Fig. 3f), which allows for locating positions of the silver nanowires. Although the emission intensity of nanocrystals placed away from the nanowires is considerably less than for nanocrystals on a glass substrate (in agreement with the results shown in Fig. 3b), we find strong enhancement of emission for those NCs, which are located in the close vicinity of silver nanowires. In fact, the images shown in Fig. 3c,d are qualitatively similar, except for the intensities. The average luminescence intensity of NCs reaches about 60 kcps for both emissions and is thus significantly higher (fourfold) than for NCs on graphene, where no NWs were present. We conclude, that despite the energy transfer to graphene, the effect of metal-enhanced fluorescence is still capable of increasing the emission of individual NCs. The influence of the energy transfer is visible as narrowing of the intensity distribution width (equal to about 80 kcps). As discussed above, this behavior is characteristic for graphene-coupled emitters, and it indicates that interaction between nanocrystals and graphene still occurs, despite the presence of nanowires.

Experiments carried out in the steady-state mode, while providing a way to compare the intensities and thus enabling estimation of the emission enhancement, fall short in regard to assess whether enhanced absorption or emission rates are responsible for this enhancement. It is also difficult to conclusively demonstrate and quantify interactions of NCs with graphene, as well as NCs and NWs with graphene in the three-component hybrid nanostructure. These questions can be answered by measuring luminescence decays of individual nanocrystals. Again, due to the absence of graphene photoluminescence, we focused solely on determining the optical response of the sensitizers (nanocrystals).

**Figure 5:**
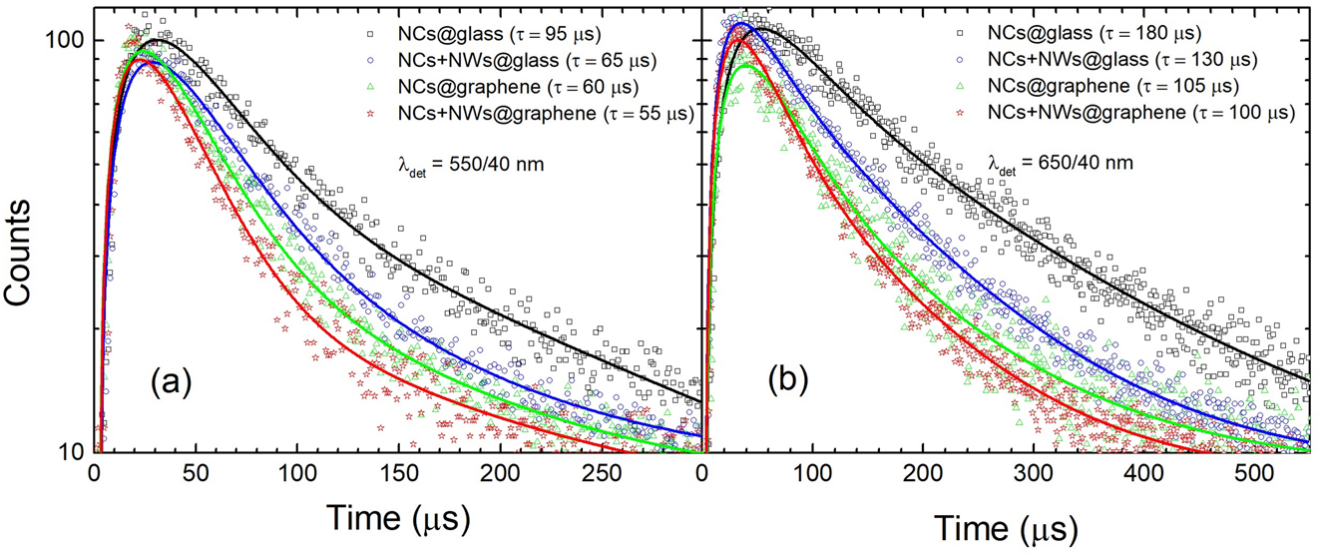
Luminescence transients of (**a**) green and (**b**) red emission of the NCs. Black and green lines/points correspond to NCs on glass and graphene, respectively, while blue and red lines/points correspond to NCs combined with NWs placed on glass and graphene, respectively.

**Figure 6:**
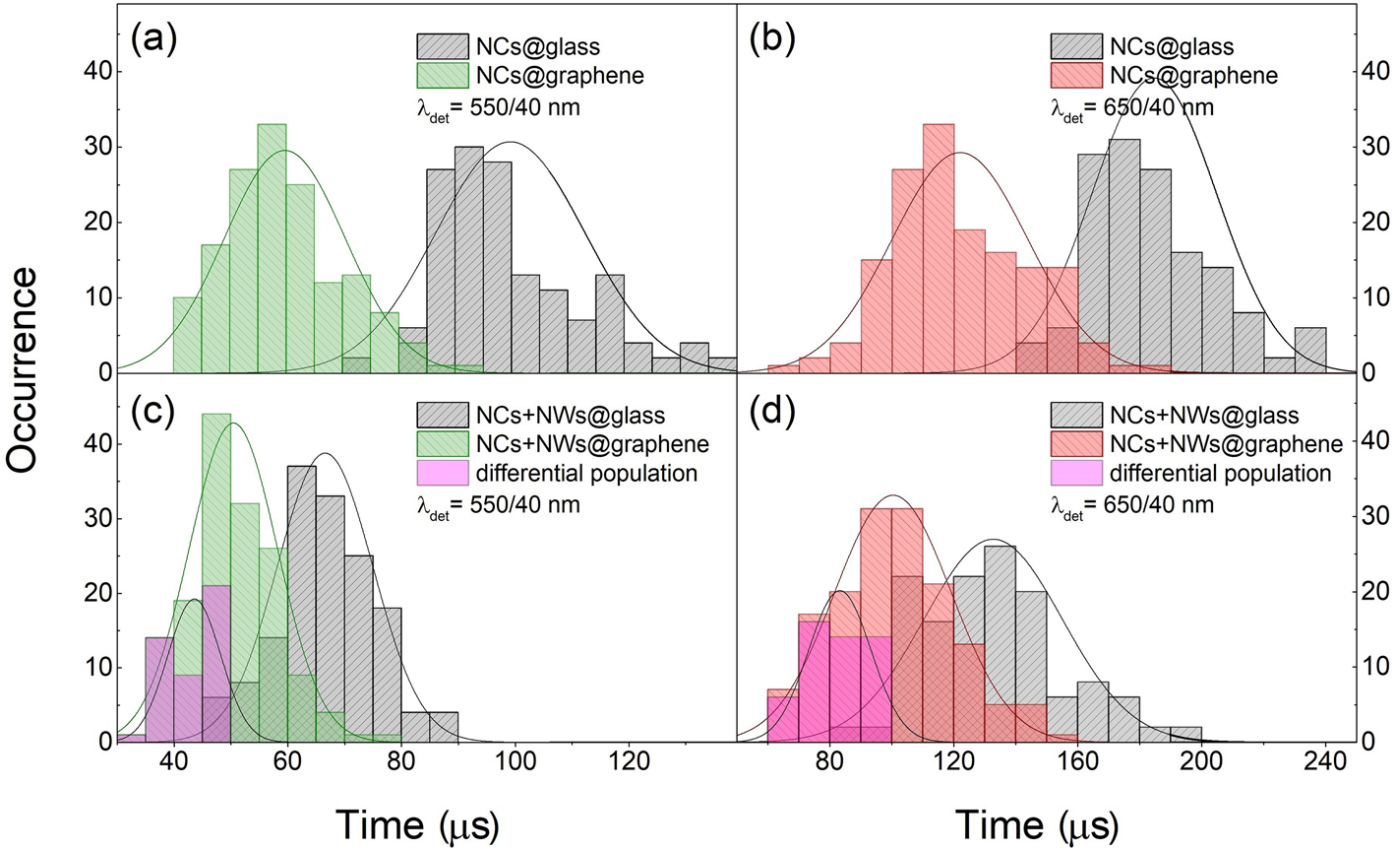
Histograms of luminescence decay times, acquired for: (**a**,**b**) nanocrystals and (**c**,**d**) nanocrystals coupled with silver nanowires, deposited on glass and graphene. Pink bars denote residual population of NCs coupled to graphene and silver NWs. Each distribution contains about 150 experimental points. Decay times reflect intensity-weighted average lifetimes^26^.

In Fig. 5 we compare typical luminescence transients measured for individual nanocrystals for both studied spectral regions: (a) 550 nm, and (b) 650 nm. Blue, green, and red points correspond to the decays measured for NCs coupled to silver nanowires deposited on glass, for NCs deposited on graphene, and for NCs deposited on graphene and coupled to a silver nanowire, respectively. Black points represent the reference, which are NCs on a glass substrate. It can be seen that for each measured nanocrystal the decay curves feature multi-exponential character^21^. The origin of this behavior is related to spatial distribution of Er^3+^ ions within nanocrystals, resulting in variation of distances between particular ions and nanowire and/or graphene. In general, we observe monotonic shortening of emission lifetimes in the direction from NCs coupled with NWs (on glass), through those coupled with graphene, till the NCs coupled with NWs on graphene. As displayed in Fig. 5, the experimentally measured decay curves are quite well reproduced by bi-exponential fits (solid lines), which may suggest existence of two populations of Er^3+^ ions, localized closer and further from the surface of the nanocrystal. In order to simplify the analysis, we extracted decay parameters using the approach based on amplitude-weighted average decay time^27^. The results of this analysis are summarized in Fig. 6.

According to the statistical analysis, the average decay time estimated for NCs on glass (reference) is about 100 μs for green, and 180 μs for red emission (Fig. 6a,b). For the NCs deposited on graphene a significant shift of the lifetime distribution towards shorter lifetimes is observed, with average decay times of about 60 μs for green and 120 μs for red emission (Fig. 6a,b). This, accompanied by already discussed quenching of the emission intensity (Fig. 4a,b), is a clear indication of the non-radiative energy transfer from NCs (Er^3+^ ions) to graphene monolayer. Interestingly, very similar values of the decay times we extracted for NCs coupled with NWs on glass. The average lifetimes are reduced to 65 μs for green and 130 μs red emission (Fig. 6c,d). This shortening, however, is a consequence of enhanced radiative emission rates of NCs placed the vicinity of NWs, as confirmed by increase of the luminescence intensity (Fig. 4c,d).

According to acquired data we noticed inhomogeneous character of the samples. This is due to inevitable distribution of nanocrystals sizes, nanowires diameters/lengths, as well as random deposition of these nanostructures on the glass/graphene surface. Random distances between interacting objects strongly determine strength of the interactions, what can blur the image of physical processes taking place within considered system. For this reason, to draw more universal conclusions, we accomplished statistical analysis of estimated lifetimes and presented in histograms in Fig. 6a,c (green emission), Fig. 6b,d (red emission).

We observed quantitatively new effects for three-component sample. Since it consists of NCs, NWs and graphene, one would expect much more complex interactions occurring between its ingredients than already discuss. As presented in Fig. 6c,d, histograms of lifetime distributions are distinctly shifted towards shorter times with respect the previous results, and centered at 50 μs and 100 μs for green and red emission, respectively. This demonstrates that three-component sample behave different than already discussed cases, so NCs on graphene or NCs-NWs on glass. Therefore, due to random character of nanoparticles arrangement, these histograms need to be corrected. Statistically, only some NCs are localized in the manner that enables simultaneous interaction with NWs and graphene. This is more likely to find NCs interacting only with graphene or only with silver nanowires. For this reason, to expose the presence of NCs-NW-graphene-coupled hybrids, we subtracted from these histograms distributions received for NCs on graphene and NCs-NWs on glass. The residual distribution is presented in Fig. 6c,d (pink bars), where negative, non-physical occurrences are omitted. Indeed, the residual population feature very short decay times, about 40 μs for green and 80 μs for red emission on average, which we attribute strictly to three-components hybrid nanostructures. Observed distortion from pure mono-exponential profile increases in the order NCs on glass, NCs on graphene, NCs-NWs on glass, and NCs-NWs on graphene (Fig. 5a,b). This observation additionally proves that NCs can interact with graphene and NWs simultaneously, forming new nanostructure combining bright and stable up-conversion luminescence of NCs with excellent electrical, thermal and mechanical properties of graphene.

## Conclusions

In this work we comprehensively describe the optical properties of a three-component hybrid nanostructure consisting of graphene, up-conversion nanocrystals and silver nanowires. For nanocrystals on graphene, where the energy transfer is dominant, we observe decrease of the emission intensity accompanied by shortening of luminescence decay times. The results of single nanocrystal spectroscopy prove that introducing silver nanowires yields strong increase of quantum efficiency and thus the emission intensity, despite the quenching due to the energy transfer. Indeed, fourfold intensity enhancement for single nanocrystals coupled with silver nanowires can be achieved. Such a strong enhancement emerges despite simultaneous interaction with graphene. Importantly, the acquired luminescence decay profiles confirm that nanocrystals can simultaneously interact with both graphene and silver nanowires. The hybrid nanostructure described in this work provides a way to combine optical activity of up-conversion nanocrystals and enhancement provided by metallic nanowires with excellent electrical and mechanical properties of graphene. The results obtained the combination of nanomaterials with different properties (up-conversion luminescence, energy transfer, and plasmonic interactions), will help to extend fundamental understanding of the physical interactions between these and other rather different species (including semiconductors, dielectrics and metals). Moreover, these functionalities can be utilized for sensing, imaging, or in diagnostic applications.

## Supplementary Information

Supplementary Information
