# Supplementary material for: Silver nanowires as plasmonic compensators of luminescence quenching in single up-converting nanocrystals deposited on graphene
Source: Sci Rep. 2021 Feb 11;11:3557. doi: 10.1038/s41598-021-82699-y (PMC7878765; doi:10.1038/s41598-021-82699-y)
Supplement: Supplementary file 1 — Supplementary Information [file 41598_2021_82699_MOESM1_ESM.docx]

**SUPPORTING INFORMATION FOR**

**Silver nanowires as plasmonic compensators of luminescence quenching in single up-converting nanocrystals deposited on graphene**

A. Prymaczek^1^, M. Ćwierzona^1^, M. A. Antoniak^2^, M. Nyk^2^, S. Mackowski^1^, D. Piatkowski^1*^

## ^1^ Institute of Physics, Faculty of Physics, Astronomy and Informatics,

## Nicolaus Copernicus University, Grudziądzka 5, 87-100 Toruń, Poland

## ^2^ Advanced Materials Engineering and Modelling Group, Faculty of Chemistry,

## Wroclaw University of Technology, Wybrzeże Wyspiańskiego 27, 50-370 Wroclaw, Poland

* dapi@fizyka.umk.pl

(a) (b) (c)


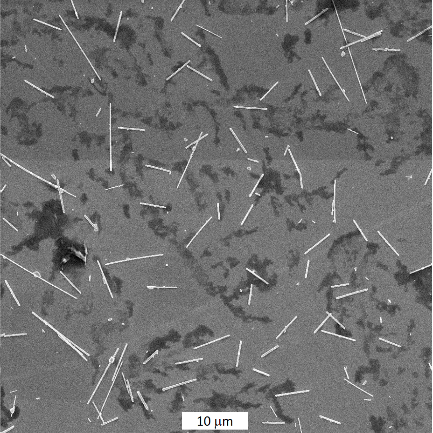

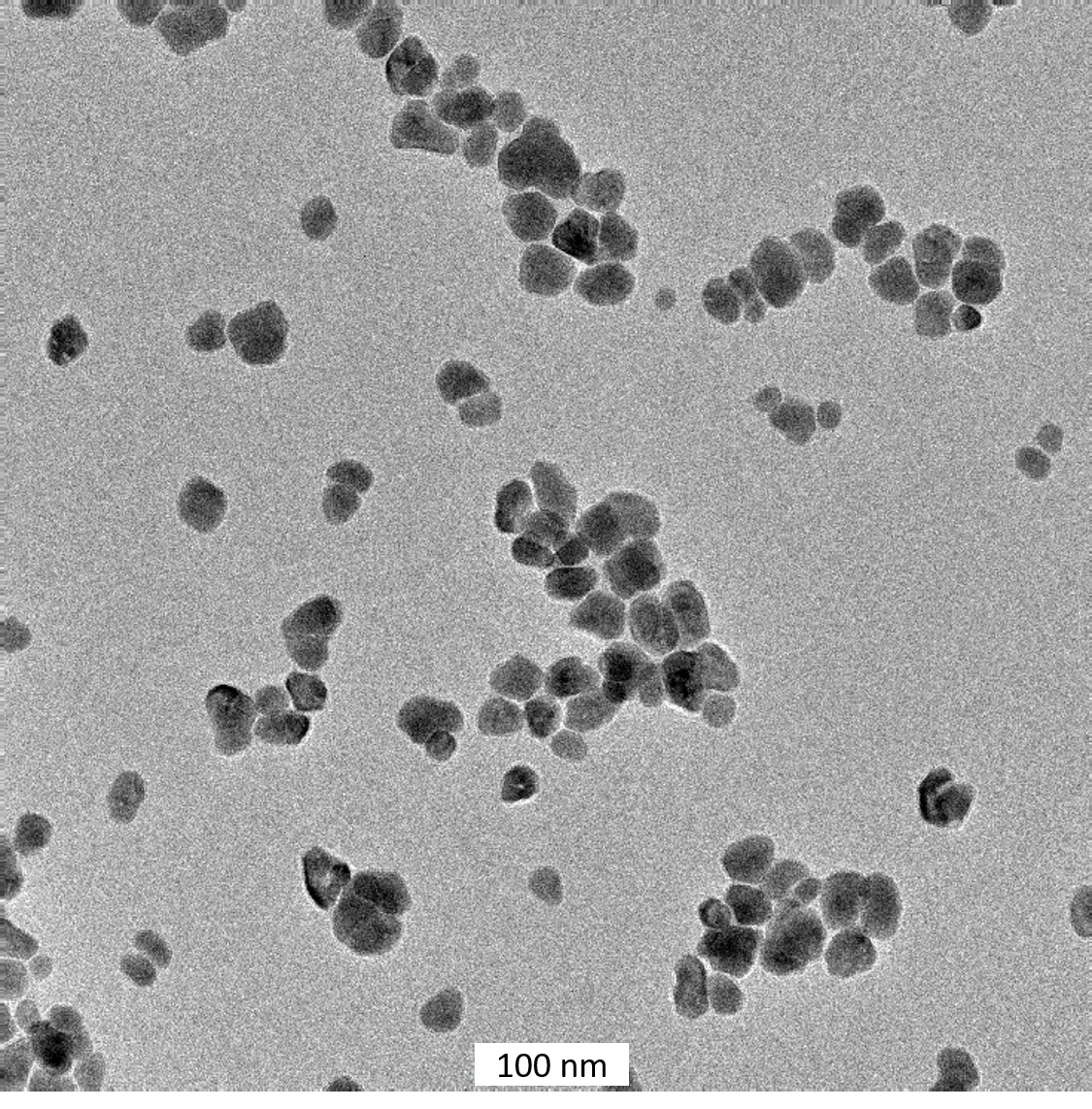

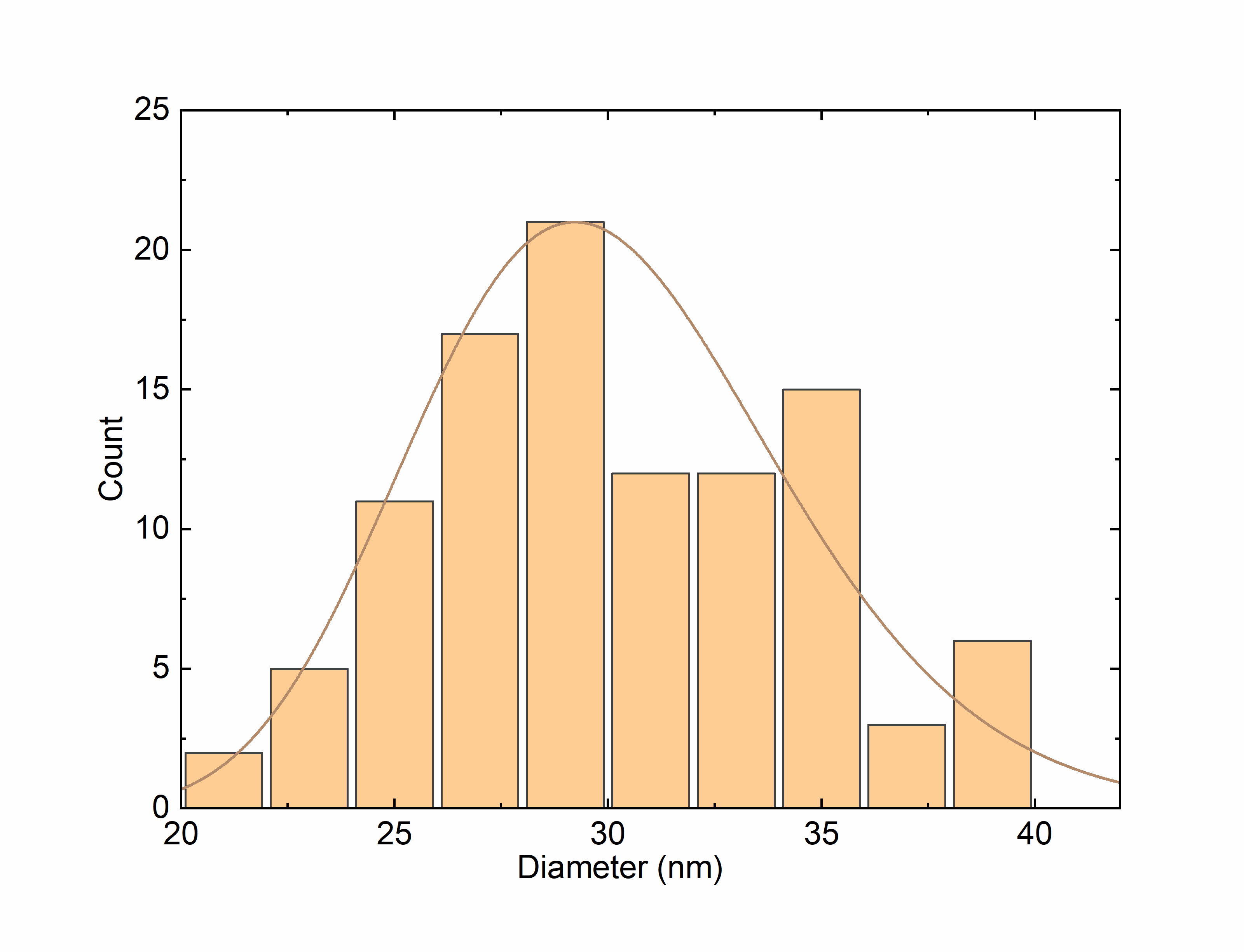


Fig. S1. (a) SEM image of silver nanowires. (b) TEM image of NaYF_4_:Er^3+^/Yb^3+^ nanocrystals, and (c) diameter distribution of single NaYF_4_:Er^3+^/Yb^3+^ nanocrystals.

1. (b) (c)


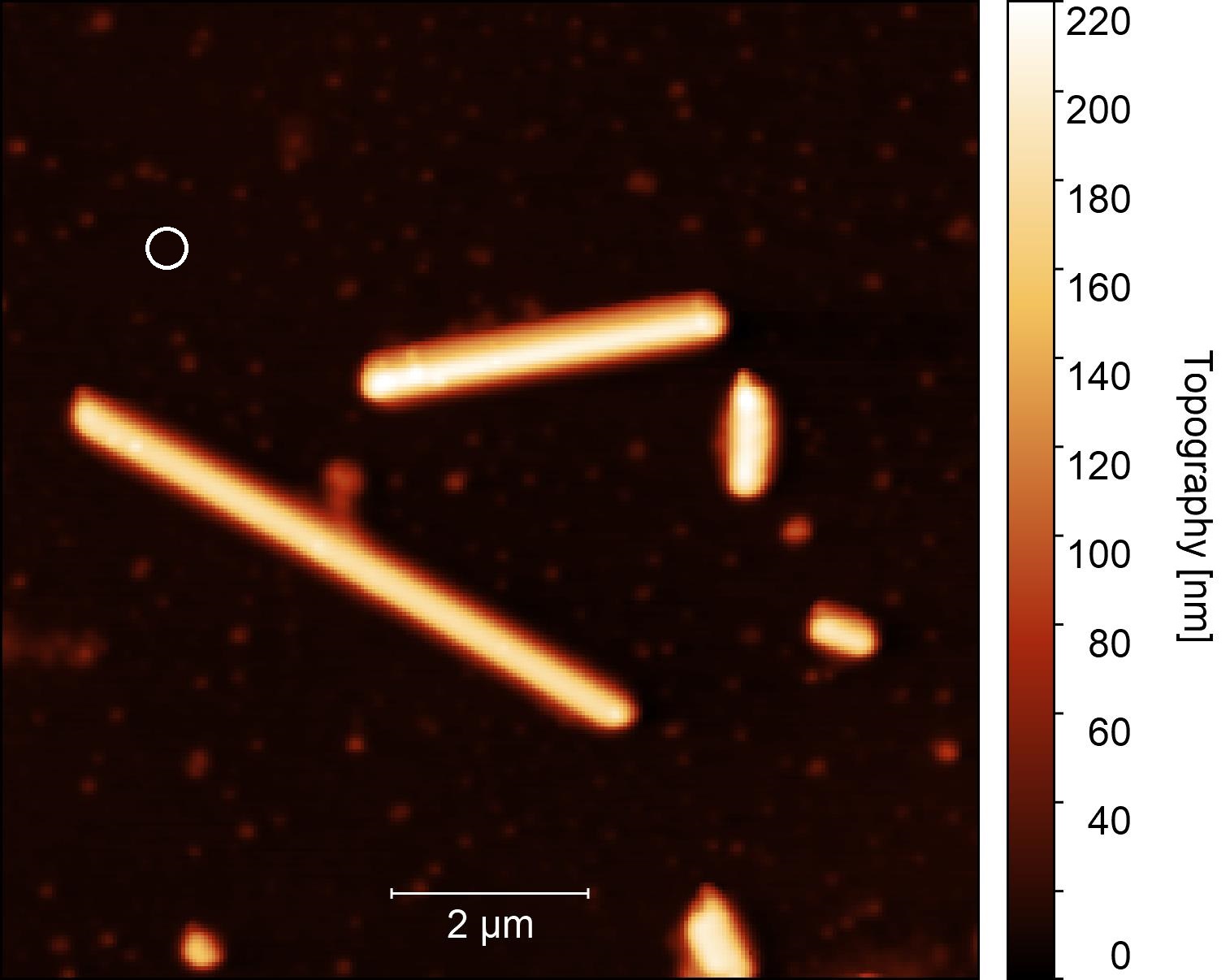

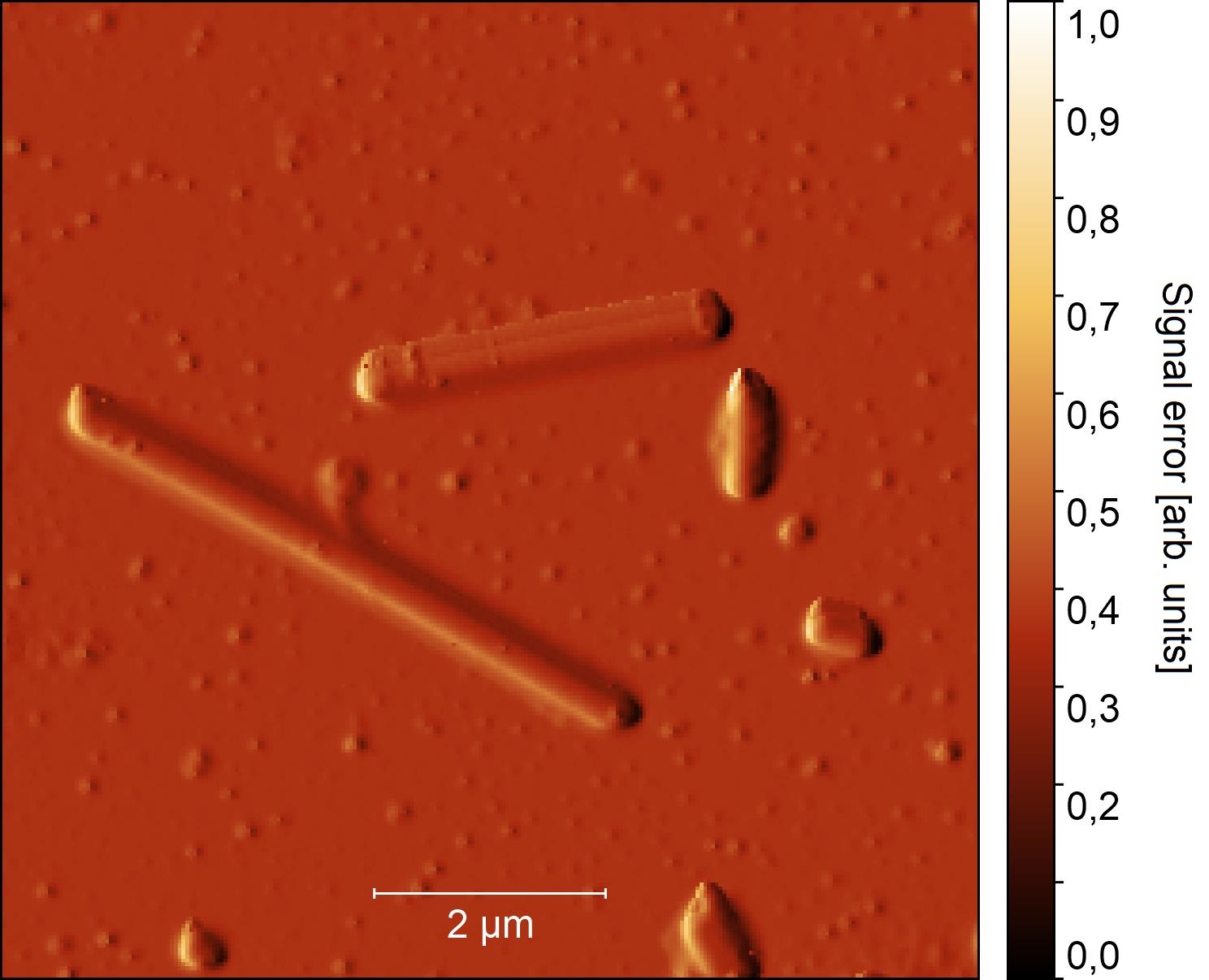

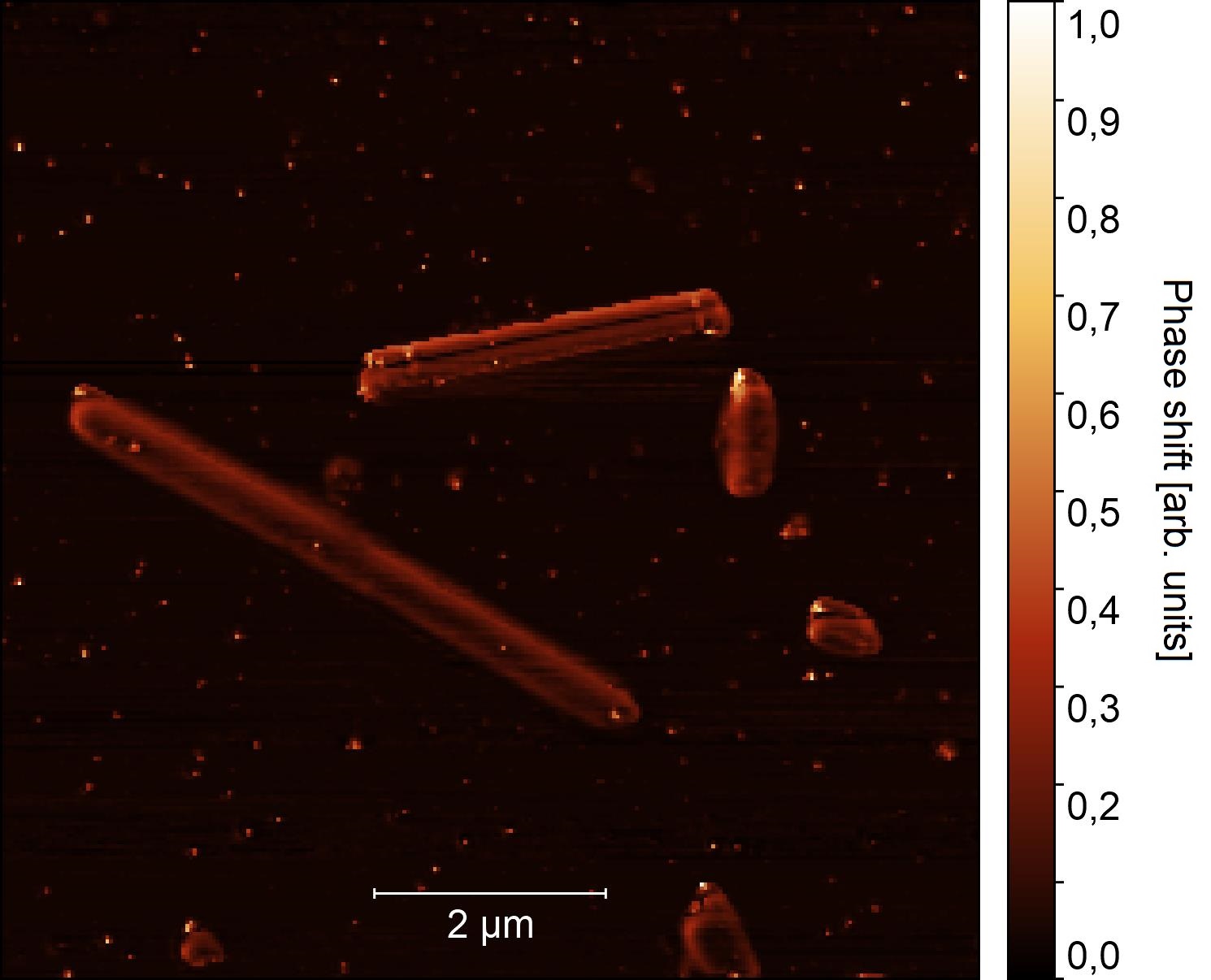


Fig. S2. Morphology of a typical sample. NaYF_4_:Er^3+^/Yb^3+^ nanocrystals and silver nanowires deposited on glass substrate, visualized by an AFM microscope. Topography, signal error, and the phase shift images are presented in sections (a), (b) and (c), respectively. White circle in picture (a) represents the size of the laser spot (λ=980 nm), focused by the microscope objective of NA= 1.49.
